# Sex-Dependent Effects of Dietary Genistein on Echocardiographic Profile and Cardiac GLUT4 Signaling in Mice

**DOI:** 10.1155/2016/1796357

**Published:** 2016-07-04

**Authors:** Lana Leung, Joshua B. Martin, Todd Lawmaster, Kathryn Arthur, Tom L. Broderick, Layla Al-Nakkash

**Affiliations:** ^1^Department of Physiology, Midwestern University, 19555 N. 59th Avenue, Glendale, AZ 85308, USA; ^2^Barrow Neurological Institute, St. Joseph's Hospital and Medical Center, 350 West Thomas Road, Phoenix, AZ 85013, USA

## Abstract

This study aimed to determine whether genistein diet resulted in changes in cardiac function, using echocardiography, and expression of key proteins involved in glucose uptake by the myocardium. Intact male and female C57BL/6J mice (aged 4–6 weeks) were fed either 600 mg genistein/kg diet (600 G) or 0 mg genistein/kg diet (0 G) for 4 weeks. Echocardiography data revealed sex-dependent differences in the absence of genistein: compared to females, hearts from males exhibited increased systolic left ventricle internal dimension (LVIDs), producing a decrease in function, expressed as fractional shortening (FS). Genistein diet also induced echocardiographic changes in function: in female hearts, 600G induced a 1.5-fold (*P* < 0.05) increase in LVIDs, resulting in a significant decrease in FS and whole heart surface area when compared to controls (fed 0 G). Genistein diet increased cardiac GLUT4 protein expression in both males (1.51-fold, *P* < 0.05) and females (1.76-fold, *P* < 0.05). However, no effects on the expression of notable intracellular signaling glucose uptake-regulated proteins were observed. Our data indicate that consumption of genistein diet for 4 weeks induces echocardiographic changes in indices of systolic function in females and has beneficial effects on cardiac GLUT4 protein expression in both males and females.

## 1. Introduction

Genistein, a naturally occurring isoflavonic phytoestrogen, is found in high concentrations in soy products [1]. Genistein binds to estrogen receptors, and while it exhibits a similar potency to 17*β*-estradiol, it has been shown be a natural alternative to estrogen resulting in noted improvements in cardiovascular health [2], without undesirable effects on the female reproductive system [3], or incidence of cancer [4]. Several health benefits for humans consuming soy-based foods have been established in recent years, including a reduced risk of age-related cardiovascular and coronary artery disease [2, 5–10].

Cardiovascular disease is a major cause of morbidity in the developed world, and moreover, the risk in women is significantly increased during postmenopause [11]. Thus, in order to determine the metabolic underpinnings explaining reduced cardiovascular heath in postmenopausal women, several studies using well-established rodent models of postmenopause have assessed the effects of genistein as an alternative to estrogen replacement on cardiac function in ovariectomized (OVX) mouse models. For example, using OVX C57BL/6J mice, Nguyen et al. [12] demonstrated a beneficial genistein dose-dependent effect on cardiac mass and expression of genes involved in Akt signaling and cardiomyocyte protection. Given the prevalence of cardiovascular disease types seen clinically, other studies have utilized common models of left ventricular (LV) dysfunction and assessed the benefits of supplemental genistein thereon. Genistein administered subcutaneously daily for 10 days has been shown to increase oxytocin receptors with an associated improvement in LV fractional shortening and decreased blood pressure in OVX spontaneously hypertensive rats [13]. Recently, Qin et al. [14] examined the effects of an 8-week administration of genistein (via daily gavage) on a murine model of LV pressure overload-induced cardiac fibrosis, induced by transverse aortic constriction, with genistein showing improvement in cardiac function and inhibition of cardiac fibrosis. In the ovariectomized rat, we have shown that a 2-day treatment by subcutaneous injections improves LV function and provides myocardial protection against ischemia and reperfusion in the isolated working heart [15]. Extending treatment with genistein to a period of 2 weeks in the ovariectomized rat resulted in decreased body weight and aortic pulse pressure, induction of a bradycardic effect, and decreased insulin resistance [16]. In the absence of ovariectomy (i.e., in intact mice), feeding mice a genistein-containing diet for a period of 8 weeks improved cardiovascular risk factors and aortic reactivity and produced favorable effects on glucose metabolism by increasing the expression of GLUT4 in the myocardium [17]. Taken together, our previous studies indicate that supplemental genistein, regardless of the route of administration, has benefits on cardiovascular function. Of interest is the increase in GLUT4 translocation in the myocardium, because previous studies have shown that improving glucose metabolism in the failing or insulin-resistant heart is associated with cardioprotection [18]. However, the mechanism by which GLUT4 translocation is enhanced with genistein treatment has not been addressed in a disease- and stress-free heart. Much less is known about the chronic effects of dietary genistein in intact healthy male and female mice. To our knowledge there have been no studies to date aimed at examining the combined effects of genistein on* in vivo* cardiac function and structure, in addition to protein expression relating to glucose uptake in healthy mice. Thus, the purpose of the present study was to examine the effects of dietary genistein treatment on cardiac function utilizing echocardiography, an important noninvasive tool that provides* in vivo* measurement of systolic and diastolic function. Furthermore, the effect of genistein diet on the expression of key proteins involved in the translocation of GLUT4 through insulin signaling mechanisms in cardiac tissue was determined.

## 2. Methods

### 2.1. Mice and Diet Study

C57BL/6J female and male intact mice were purchased from Jackson Laboratory (Bar Harbor, ME), at 4–6 weeks of age and randomly assigned to the following diet groups: 600 G, (600 mg/kg diet genistein) or control 0 G (0 mg/kg diet genistein) [19]. Casein-based diets were specially formulated, as per our previous studies [19], and purchased from Dyets Inc. (Bethlehem, PA). Animals were housed in an animal care facility, two per cage, with a 12 : 12-hour light-dark cycle and given food (genistein-free diet, 0 G, or genistein-containing diet, 600 G) and water* ad libitum*. Body weight was measured weekly during the study and general health was monitored biweekly. Mice were maintained on diets for a period of 4 weeks. At the end of the diet study, mice were asphyxiated in an atmosphere of 100% CO_2_, followed by surgical thoracotomy to induce pneumothorax. Whole hearts were immediately harvested, snap-frozen in liquid nitrogen, and stored at −80°C until use. Animal experimental protocols were approved by the Midwestern University Animal Care and Use Committee.

### 2.2. Echocardiographic Assessment of Heart Function

At the end of the 4-week diet study, echocardiography was assessed using an Acuson Sequoia 512 ultrasound machine, utilizing methods described previously [20, 21]. Mice were anesthetized via intramuscular injection of xylazine (5 mg/kg) and ketamine (100 mg/kg) and kept on a heating pad throughout the experiment. Echocardiograms were obtained from the anesthetized mice, in a supine position. A 14 MHz linear transducer was applied to the left hemithorax, with a layer of ultrasonic transmission gel applied between the mouse and transducer to obtain the following measures: diastolic interventricular wall thickness (IVSd), systolic interventricular wall thickness (IVSs), diastolic LV internal dimension (LVIDd), systolic LV internal dimension (LVIDs), diastolic LV posterior wall thickness (LVPWd), and systolic LV posterior wall thickness (LVPWs), and the percent fractional shortening (FS%) was calculated using the formula ([LVIDd − LVIDs]/LVIDd*∗*100). All data are expressed as the average of at least 2 separate scans. Echocardiographic measurements were completed in a random and blinded fashion. Following completion of the echocardiographic measures, mice were maintained in a heated environment and were monitored until being fully recovered prior to return to their cages.

### 2.3. Measurement of Cardiac Collagen

Collagen concentration was determined as described previously via quantification of the collagen-specific amino acid hydroxyproline, HYP [22]. Briefly, hearts were submerged in 6 M HCl, hydrolyzed at 100°C for 24 hours, then neutralized with 6 M NaOH, and filtered (0.2 *μ*m filter). Samples were titrated to pH 8–13 using 6 M NaOH or 6 M HCl. Samples were derivatized, and high performance liquid chromatography was utilized (Shimadzu Scientific Instruments, Prominence, Columbia, MD). Separation of HYP was mediated via a Waters XTerra RP18 column (Waters Corporation, Milford, MA) using a methodology with a flow rate of 1.0 mL/min (mobile phase: 35% acetonitrile, ~5% sodium acetate, and ~2% acetic acid). Column temperature was set at 25°C (Shimadzu Scientific Instruments, CTO-20AT, Columbia, MD, USA). Fluorescence was monitored at 260 nm excitation/316 nm emission (Shimadzu Scientific Instruments, RF-20A, Columbia, MD). Peaks were integrated using chromatography software (Shimadzu LC Solution, Shimadzu Scientific Instruments, Prominence, Columbia, MD).

### 2.4. Cardiac Protein Expression

Standard western blot techniques, as described previously [17], were utilized to measure the following proteins: GLUT4 (~40–48 kD), iNOS (~130 kD), eNOS (~130 kD), SYNIP (~62 kD), Akt (~60 kD), AMPK (~62 kD), and MEF2a (~55 kD). Total protein concentration of whole heart tissue was determined (Pierce, Rockford IL). In brief, sample was loaded on 10% Bis-Tris gels (GLUT4), 10% Tris-Glycine gels (iNOS and eNOS), or 4–12% Tris-Glycine gels (SYNIP, Akt, AMPK, MEF2a) and ran at either 200 V (Glut4) or 100 V (iNOS and eNOS) for ~50 min or ran at 150 volts (SYNIP, Akt, AMPK, and MEF2a) for ~90 min. Gels were transferred at 100 V for 40 min (GLUT4) or 30 V for 120 min (iNOS, eNOS, SYNIP, Akt, AMPK, and MEF2a), at 4°C. Blots were incubated with primary antibody (1 : 5000 anti-GLUT4 from rabbit; 1 : 500 anti-iNOS or anti-eNOS), SYNIP (1 : 5000), Akt (1 : 1000), AMPK (1 : 1000), and MEF2A (1 : 5000) overnight at 4°C in 5% milk in phosphate buffered saline solution + 0.1% Tween (PBST). Blots were then incubated with secondary antibody (1 : 1200–1 : 10,000 anti-rabbit IgG, Amersham Biosciences, Piscataway, NJ) in 3% milk in PBST for 1 hour at room temperature. To reprobe for GAPDH or actin, blots were incubated with anti-GAPDH or anti-actin primary antibody (1 : 4000) for 60 min at room temperature and washed and incubated with secondary anti-mouse IgG HRP-linked antibody (1 : 7500). Proteins were visualized with enhanced chemiluminescent substrate (Amersham Biosciences, Piscataway NJ), and density of bands was measured using Image J (NIH, Bethesda, MD).

The following antibodies were purchased: anti-GLUT4 (Calbiochem, Gibbstown, NJ), anti-iNOS and anti-eNOS (Santa Cruz Biotechnology, CA), SYNIP, Akt, AMPK, and anti-mouse IgG HRP-linked antibody (Cell Signaling, Danver, MA), MEF2A (Abcam, Cambridge, MA), anti-GAPDH primary antibody (Sigma-Aldrich, St. Louis, MO), anti-actin primary antibody (Clone C4, Millipore, Billerica, MA).

### 2.5. Cardiac Morphology

For heart morphometric analyses, each whole heart was embedded in Tissue Tek Optimal Cutting Temperature Compound (OCT, Sakura Finetek, Torrance, CA), and the method used was as described previously [23]. Briefly, sections (10–12 *μ*M thickness) were treated with hematoxylin and then rinsed with water, Scott's Solution, 95% and 200% ethanol, and eosin, followed by Histo-Clear (National Diagnostics, Atlanta, GA). Measures were taken of whole heart surface area and left ventricle surface area. Area assessments were made with 100x objective (an average of five measures taken per heart was used).

### 2.6. Statistics

Statistical analysis was performed using Graphpad Prism 4 (Graphpad Software Inc., San Diego, CA). All values are reported as mean ± SD. A one-way ANOVA, followed by Newman-Keuls comparison for post hoc analysis, was used to determine differences between all four diet groups. Significance is shown as *P* < 0.05.

## 3. Results

### 3.1. Echocardiography

Table 1 demonstrates the effects of a 4-week genistein diet on echocardiographic parameters of male and female mice. We found in female mice a genistein-dependent increase (1.48-fold) in systolic left ventricle internal dimension (LVIDs), with a concomitant significant 44% decrease in fractional shortening (FS). Interestingly, LVIDs was significantly greater (1.56-fold) in male controls than in females and FS was significantly decreased (by 43%) in males controls compared to female control counterparts. Despite the changes in FS, values remained within the typical range in each sex [21]. There were no changes in any of the other parameters measured.

### 3.2. Cardiac Structure and Collagen Content

**Figure 1:**
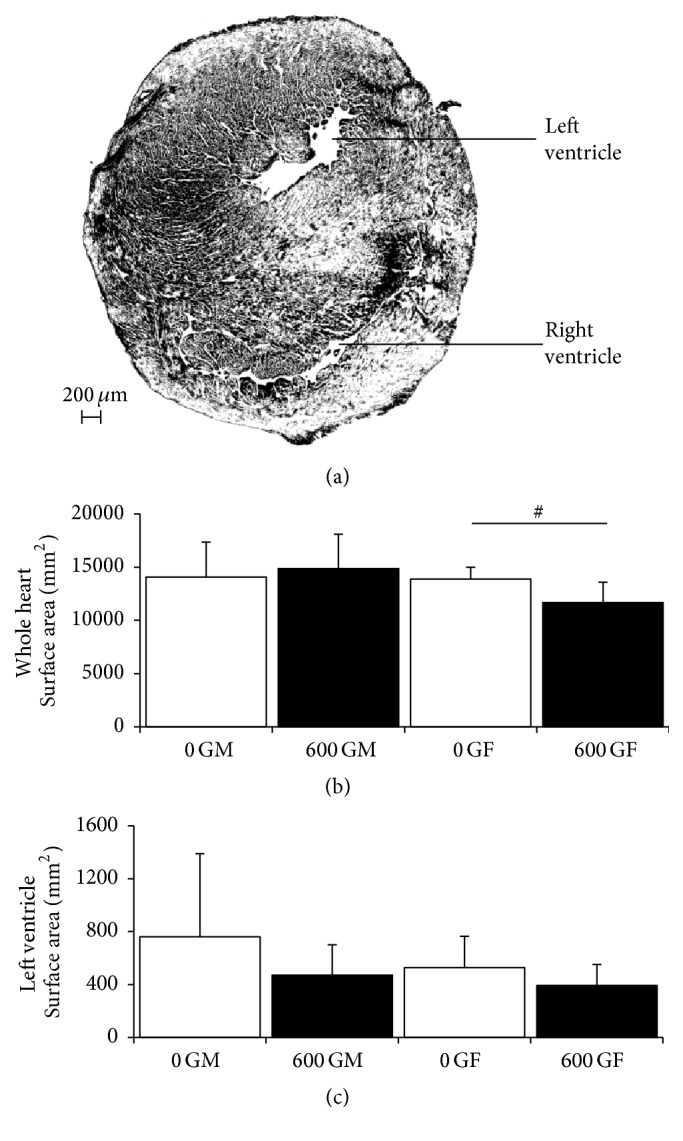
Effect of genistein treatment on whole heart surface area and left ventricle surface area in intact male and female mice. (a) Representative histological section of cardiac tissue. This image is from a 0 G fed male. Right and left ventricle spaces are indicated. (b) Average whole heart surface area. *n* = 4–6/group. (c) Average left ventricle surface area. *n* = 4–6/group. 0 GM: genistein-free fed males; 600 GM: genistein-fed males; 0 GF: genistein-free fed females; 600 GF: genistein-fed females. The genistein-free fed groups (0 G) are represented by open bars, and genistein-fed groups (600 G) are represented by the solid bars. Values are reported as mean ± SD. # denotes genistein-mediated difference, *P* < 0.05.

**Figure 2:**
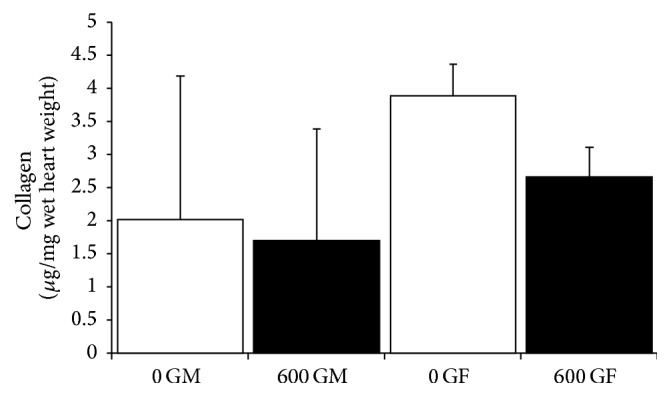
Effect of genistein treatment on cardiac collagen content in intact male and female mice. Cardiac collagen content normalized to cardiac wet weight is shown. 0 GM: genistein-free fed males; 600 GM: genistein-fed males; 0 GF: genistein-free fed females; 600 GF: genistein-fed females. The genistein-free fed groups (0 G) are represented by open bars, and genistein-fed groups (600 G) are represented by the solid bars. Values are reported as mean ± SD. *n* = 4–6/group.

Sections of whole cardiac tissue were assessed for whole heart surface area and LV surface area (Figure 1(a)). In female mice, consumption of a genistein diet for 4 weeks resulted in a significant decrease in whole heart surface area (0 GF = 13883.5 ± 1103.0 *μ*m^2^ (*n* = 5) and 600 GF = 11677.1 ± 1903.0 *μ*m^2^ (*n* = 6), *P* < 0.05, Figure 1(b)). There was a trend for a concomitant genistein-mediated decrease in LV surface area in females. There was no effect in male mice (Figure 1(c)). Cardiac collagen content was modified neither by genistein diet nor by sex (Figure 2).

### 3.3. Cardiac Protein Expression

**Figure 3:**
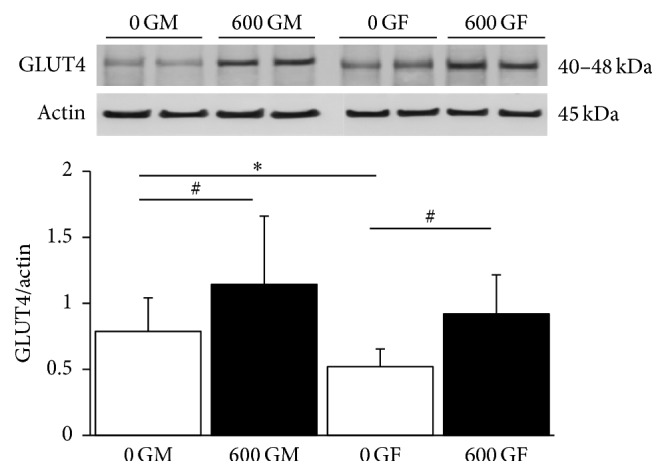
Effect of genistein treatment on total cardiac GLUT4 protein expression in intact male and female cardiac tissue. Typical western blot demonstrating GLUT4 and actin expression in cardiac tissue from two samples each of 600 G- and 0 G-treated male and female mice. Average GLUT4/actin ratio is shown comparing 600 G- and 0 G-fed male and female mice (*n* = 10/group). GLUT4 and actin bands were observed at 40–48 kDa and 43 kDa, respectively. The genistein-free fed groups (0 G) are represented by open bars, and genistein-fed groups (600 G) are represented by the solid bars. Values are reported as mean ± SD and *∗* denotes sex-dependent difference (*P* < 0.05). # denotes genistein-mediated difference, *P* < 0.05. 0 GF: genistein-free fed females; 0 GM: genistein-free fed males; 600 GF: 600 mg genistein/kg diet-fed females; 600 GM: 600 mg genistein/kg diet-fed males; GLUT4: glucose transporter 4.

We first determined total GLUT4 content in hearts from mice fed genistein for a period of 4 weeks. As shown in Figure 3, feeding mice a genistein-containing diet increased the expression of total GLUT4, confirming our earlier findings [17]. Western blot analysis revealed that cardiac GLUT4 expression was significantly increased in both male (1.51-fold from 0.79 ± 0.25 (*n* = 10) to 1.18 ± 0.51 (*n* = 9), *P* < 0.05) and female (1.76-fold from 0.52 ± 0.13 (*n* = 10) to 0.92 ± 0.29 (*n* = 10), *P* < 0.05) mice fed 600 G. Interestingly, male control mice (0 GM) had significantly greater GLUT4 expression (1.52-fold) compared to females control counterparts.

**Figure 4:**
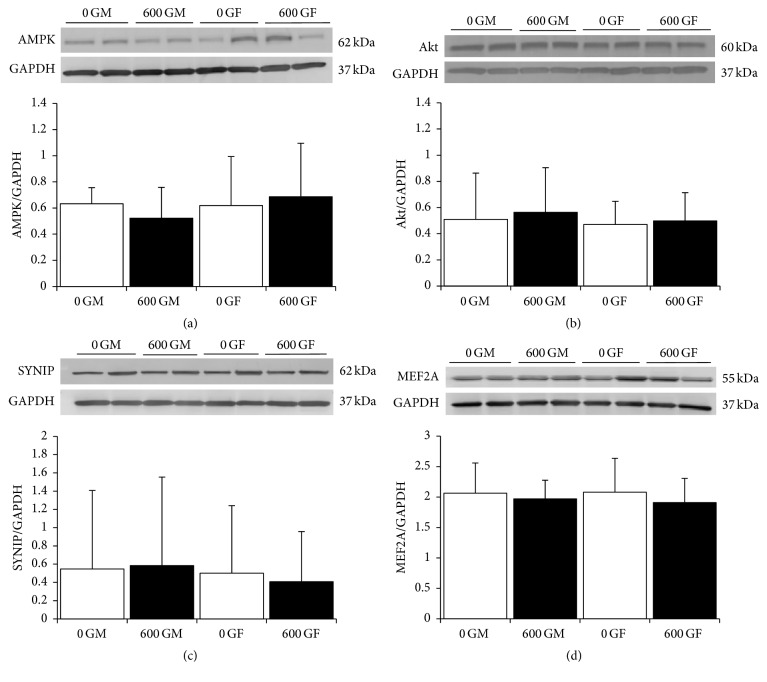
Effect of genistein treatment on total cardiac Akt, AMPK, SYNIP, and MEF2A protein expression in intact male and female mice. (a) AMPK. Typical western blot demonstrating AMPK and GAPDH expression in cardiac tissue from two samples each of 600 G- and 0 G-treated male and female mice. Average AMPK/GAPDH ratio is shown comparing 600 G- and 0 G-fed male and female mice (*n* = 6/group). AMPK and GAPDH bands were observed at 62 kDa and 37 kDa, respectively. (b) Akt. Typical western blot demonstrating Akt and GAPDH expression in cardiac tissue from two samples each of 600 G- and 0 G-treated male and female mice. Average Akt/GAPDH ratio is shown comparing 600 G- and 0 G-fed male and female mice (*n* = 8/group). Akt and GAPDH bands were observed at 60 kDa and 37 kDa, respectively. (c) SYNIP. Typical western blot demonstrating SYNIP and GAPDH expression in cardiac tissue from two samples each of 600 G- and 0 G-treated male and female mice. Average SYNIP/GAPDH ratio is shown comparing 600 G- and 0 G-fed male and female mice (*n* = 10/group). SYNIP and GAPDH bands were observed at 62 kDa and 37 kDa, respectively. (d) MEF2A. Typical western blot demonstrating MEF2A and GAPDH expression in cardiac tissue from two samples each of 600 G- and 0 G-treated male and female mice. Average MEF2A/GAPDH ratio is shown comparing 600 G- and 0 G-fed male and female mice (*n* = 6/group). MEF2A and GAPDH bands were observed at 55 kDa and 37 kDa, respectively. Values are reported as mean ± SD. 0 GF: genistein-free fed females; 0 GM: genistein-free fed males; 600 GF: 600 mg genistein/kg diet-fed females; 600 GM: 600 mg genistein/kg diet-fed males. The genistein-free fed groups (0 G) are represented by open bars, and genistein-fed groups (600 G) are represented by the solid bars.

To determine whether the genistein-mediated increase in GLUT4 content was facilitated by insulin signaling mechanisms, we measured the expression of Akt and AMPK [24, 25]. No differences in the expression of these metabolic modulators were observed between control and genistein-treated hearts, regardless of the sex of mice (Figures 4(a) and 4(b)). Since no differences were seen, we then measured the expression of downstream proteins involved in the vesicular transport of GLUT4 to the plasma membrane. As illustrated in Figures 4(c) and 4(d), expression of myocyte enhancer factor 2 (MEF2) and synip, a highly specific syntaxin-4 binding protein that regulates the docking and fusion of GLUT4-containing vesicles [26], was not significantly changed between groups.

**Figure 5:**
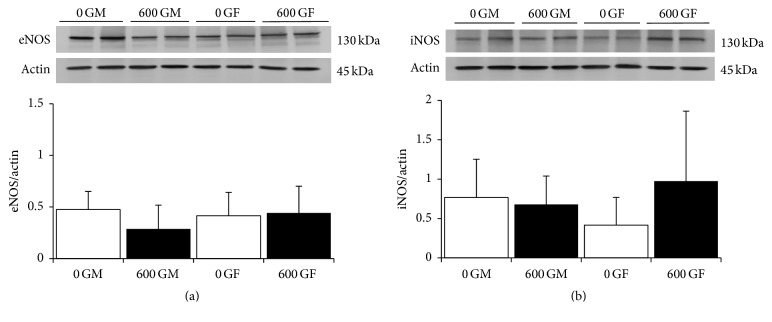
Effect of genistein treatment on total cardiac eNOS and iNOS protein expression in intact male and female mice. (a) eNOS. Typical western blot demonstrating eNOS and actin expression in cardiac tissue from two samples each of 600 G- and 0 G-treated male and female mice. Average eNOS/actin ratio is shown comparing 600 G- and 0 G-fed male and female mice (*n* = 10/group). eNOS and actin bands were observed at 130 kDa and 43 kDa, respectively. (b) iNOS. Typical western blot demonstrating iNOS and actin expression in cardiac tissue from two samples each of 600 G- and 0 G-treated male and female mice. Average iNOS/actin ratio is shown comparing 600 G- and 0 G-fed male and female mice (*n* = 10/group). iNOS and actin bands were observed at 130 and 43 kDa, respectively. Values are reported as mean ± SD; 0 GF: genistein-free fed females; 0 GM: genistein-free fed males; 600 GF: 600 mg genistein/kg diet-fed females; 600 GM: 600 mg genistein/kg diet-fed males; eNOS: endothelial nitric oxide synthase; iNOS: inducible nitric oxide synthase. The genistein-free fed groups (0 G) are represented by open bars, and genistein-fed groups (600 G) are represented by the solid bars.

Expression of eNOS, an AMPK downstream-regulated protein and marker of vascular function, was also measured. As shown in Figure 5, there were no effects of genistein or sex on the expression of eNOS and iNOS (Figures 5(a) and 5(b)), although a trend for increased iNOS expression in genistein-fed females was seen.

## 4. Discussion

We have previously reported that a diet of 600 mg genistein/kg food fed to female and male mice for a period of 4 weeks increases serum genistein levels to 7.7 ± 4.0 *μ*mol/L and 4.2 ± 2.3 *μ*mol/L, respectively [19]. Bhandari et al. [27] demonstrated that mice consuming 500 or 1000 mg genistein/kg diet generated serum genistein levels in the range of 0.5–1.5 *μ*mol/L after a period of 4 weeks on their respective diets. Thus, regular daily ingestion of this naturally occurring isoflavone by mice can maintain serum levels in a low *μ*mol/L range. Importantly, we have previously shown that female lean mice fed 600 mg genistein/kg diet for a 4-week period had significantly increased jejunal chloride secretion compared to those fed standard chow, or diets containing either 100 mg genistein/kg diet or 300 mg genistein/kg diet [19]. Thus, we chose the diet composition that we have strong evidence will yield significant effects on tissue function in mice, that is, one that is physiologically relevant and one that concomitantly relates to serum genistein levels of individuals consuming soy. Therefore, based on our treatment protocol, we predicted that consumption of a diet enriched with genistein would have beneficial effects on cardiac health in intact mice. Because we have also previously reported the occurrence of sex-dependent differences in response to genistein [19], we assessed its effects in both male and female mice.

The beneficial effect of isoflavones, including genistein, on overall cardiovascular markers and health as well as in models of LV dysfunction is well established. In mice with pressure overload-induced LV hypertrophy induced by aortic construction, genistein attenuated the development of cardiac dysfunction and hypertrophy, increased fractional shortening, and prevented the development of fibrosis [14]. In the ovariectomized spontaneously hypertensive rat, genistein significantly improved percent fractional shortening and increased the expression of oxytocin receptor content in heart [13]. A reduction in the expression of brain natriuretic peptide and fibrosis in the LV was also observed in the presence of genistein in the OVX model of estrogen deficiency [13]. In disease-free mice, however, the beneficial effects of genistein on cardiac function appear to be inconsistent, likely reflecting upon differences in the experimental conditions reported. Some studies have shown that treatment with genistein had no effect on echocardiographic indices of function in hearts from disease-free female mice [28]. Yet, herein, we report no effect of genistein on male cardiac function but a significant decrease in fractional shortening in female mice, suggesting that sensitivity to the detrimental effects of genistein may be sex-dependent, as proposed recently [28]. The exact mechanism explaining this sex effect is not clear, but it is possible that genistein, a major steroid receptor ligand, combined with endogenous estrone or estradiol levels, being higher in the plasma of female mice, may have pleiotropic biological effects of altering growth and function. Indeed, an augmented estrogenic exposure in the female mouse may lead to an early form of a dilated phenotype, indicated by an increase in LV internal volume during systole and a decrease in fractional shortening. Further, in a similar study comparing the effects of sex and supplemental genistein on heart function, a significant decrease in fractional shortening was reported in hearts from male mice exhibiting dilated cardiomyopathy as a result of a mutation in the *α*-myosin heavy chain gene treated with genistein [29]. This decrease in function in male hearts was associated with an increase in several markers of heart failure, including *β*-myosin heavy chain gene expression, fibrosis, and caspase-3 dependent apoptosis [29]. The Akt/glycogen synthase kinase 3 protein kinase pathway, which is expressed in disease [30], was also increased in male hearts. Hearts from female mice with this gene mutation also exhibited increases in *β*-myosin heavy chain gene expression and Akt/glycogen synthase kinase 3 protein kinase signaling although fraction shortening was not dramatically decreased, suggesting that these early changes may precede the development of heart failure [29]. Clearly, these differences in experimental outcomes in response to genistein treatment could be due to variances in the model used, severity of LV dysfunction, the duration of exposure to genistein, or the dose of genistein used. In addition, using fractional shortening as sole index of LV function may have limited value based on measurement of only transverse function and contractility of the inferior-lateral and interventricular septum. Further, this index may not be appropriate if any regional wall abnormalities or dilation is present, as observed in female hearts after genistein treatment [21].

Interestingly, we have previously shown that acute application of genistein improves isolated working rat heart function and induces a robust anti-ischemic effect by increasing cardiac output in ovariectomized rat hearts [15]. More recently, we have shown a genistein-mediated decrease in cardiac work (CW) based on arterial blood pressure development (of ~17%) in female mice, and since CW is an index of genistein's effect on mechanical function, we proposed that 600 G may have preferential beneficial effects on cardiac function in female mice [31].

Data from the current study indicate a genistein-induced decrease in whole heart surface area in females (with no effect in males, Figure 1(b)), in the absence of changes in heart weight (Table 1). Of note, variances in collagen content in females were greater than in male counterparts, regardless of diet group (Figure 2). Again, Qin et al. [14] have shown a genistein-induced decrease in area of fibrosis and collagen content in the aortic-constricted male mice but also found no effect of genistein on control male mice. We have previously determined a genistein-mediated increase in tendon collagen content in ovariectomized rats, but not in intact control rats [32]. Thus, literature evidence supporting a role for genistein to influence collagen content is conflicting and seemingly model and sex-dependent.

The effects of genistein on glucose uptake and thus tissue metabolism have been investigated by our lab and others. Genistein treatment has been associated with a decrease in glucose uptake in adipocytes [33–35]. A genistein-induced increase in insulin sensitivity has been reported in postmenopausal women and in male Wistar rats [36, 37] which is consistent with our previous findings in male mice using the insulin to glucose ratio as index, that is, a genistein-mediated increase in insulin/glucose ratio [31]. However, in female mice, no effect of genistein on insulin sensitivity was observed (i.e., no change in insulin/glucose ratio), which could have been due to the fact that plasma insulin levels in female mice were ~50% lower than in the male counterparts. Consistent with previously published reports of genistein's action on insulin secretion, we have demonstrated a significant 63% increase in plasma levels of insulin following consumption of genistein-containing diet (600 G) for 4 weeks in male mice [31]. The increase in circulating insulin (in males only) was accompanied by a significant 25% decrease in serum glucose levels in male mice (with no significant change noted in females) [31].

However, in addition to these differences in plasma insulin and glucose to genistein treatment, which makes it difficult to accurately assess how genistein affects glucose metabolism, we have previously demonstrated that this phytoestrogen administered for a period of 2 months increases the expression of the glucose transporter GLUT4 in male murine cardiac tissue [17]. We further confirm this finding in this current study with the significant increase in the expression of total protein GLUT4 content following genistein treatment. In the current study we show that genistein increases total GLUT4 content to the same extent in hearts from both male and female mice. Thus, it may be concluded that increases in total GLUT4 expression are observed at 4 weeks in both males and females (as in this study) and by 8 weeks in only males [17]. The genistein-mediated effects on GLUT4 expression after 4 weeks of diet are relevant to our previous determination of serum glucose levels with the same diet [31]; in males there is a genistein-mediated increase in cardiac GLUT4 expression correlating with a significant decrease in serum glucose levels, and in females there is a genistein-mediated increase in cardiac GLUT4 expression correlating with a trend for a decrease in serum glucose levels.

In order to better identify the mechanism of action for this genistein-mediated increase in GLUT4 expression, we examined by western blot analysis the expression of important intracellular regulators involved in glucose uptake. While we do not measure translocation of GLUT4, we recognize the limitations of this current study. That said, the translocation of GLUT4 from intracellular vesicles to cell surface membranes by insulin signaling mechanisms is widely known to be mediated by several pathways and involves fusogenic proteins. Activation of 5′-AMP-activated protein kinase (AMPK) via Akt is a key pathway that results in increased catabolic effects, namely, glucose uptake through the expression of GLUT4. Evidence from other studies suggests that genistein-mediated effects on GLUT4 translocation are dependent upon the animal state (i.e., normal versus inflammation). For example, in adipose tissue from normal male mice, genistein inhibits insulin-induced phosphorylation of insulin receptor substrate-1 at tyrosine residues resulting in inhibition of GLUT4 translocation; however, in mice exposed to an inflammatory stimulus, genistein then improved GLUT4 translocation [38]. In isolated male rat skeletal muscle, contraction-stimulated glucose transport was completely inhibited by genistein (acute application, 100 *μ*M), due to inhibition of glucose transporter function [39]. Moreover, in L6 myotubes (a cellular model of skeletal muscle) genistein has been shown to abolish glucose uptake [40]. In contrast, in female Zucker diabetic fatty rats treatment with genistein for 17 weeks resulted in increased GLUT4 expression in skeletal muscle [41]. Thus, based on the literature, genistein-mediated effects on GLUT4 expression and therefore on glucose regulation are likely tissue-, dose-, duration-, phenotype of the model-, and sex-specific.

Although GLUT4 content is stimulated in hearts from genistein-fed mice, this was not associated with increased expression of either AMPK or Akt. The plasma membrane translocation of GLUT4 also requires the interaction of the fusogenic plasma membrane proteins SYNIP, a SNARE-binding regulatory protein [42], and myocyte enhancer factor 2 (MEF2). Expression of both of these proteins involved in the docking and fusion of GLUT4-containing vesicles [26] was not altered in the presence of genistein. Protein expression of eNOS and iNOS, which exert important roles as an endothelium-derived vasorelaxant and in the modulation of cardiac function, respectively, and also downstream target proteins of AMPK, was not altered following treatment with genistein. Interestingly, while we did note a trend for increased iNOS expression in hearts from female-treated animals, this may explain, in part, the reduction in cardiac function seen in female hearts. Increasing evidence suggests that alterations in NO synthesis, in particular iNOS production, are of pathophysiological importance in the development of heart failure [43]. Myocardial protein expression of iNOS is increased in both volume-overload heart failure [43] and the aged mouse heart [44] contributing to depressed myocardial function. Although the exact mechanism of action of genistein on cardiac GLUT4 expression and intracellular signaling is unclear, our results provide strong evidence that the AMPK pathway is not involved in this response in hearts from healthy intact mice. Further investigation on alternate pathways and mechanisms involved in the genistein-mediated increase of this glucose transporter protein is warranted. Of note, genistein is a known activator of the cystic fibrosis transmembrane conductance regulatory protein (CFTR), a chloride channel ubiquitously expressed in epithelial tissues, and of specific interest to this study, in cardiac tissue [45]. Shuba and McDonald [46] have demonstrated that chloride current is regulated/activated by genistein in guinea pig cardiac myocytes. Thus, involvement of CFTR and potential effects of genistein diet on cardiac contractility and overall function is likely.

In conclusion, the results of this study compare for the first time the effects of genistein and sexual dimorphism on the echocardiographic profile and expression of key proteins involved in glucose uptake by the myocardium in healthy mice. In female mice, treatment with genistein increased diastolic chamber dimensions, resulting in a decrease in LV fractional shortening, although fractioning shortening was still maintained within normal limits. In the absence of genistein treatment, hearts from male mice exhibited higher diastolic chamber dimensions compared to female hearts. Although GLUT4 expression in myocardium was higher in male mice compared to female mice, the increase in the expression of this protein following dietary genistein was similar. Despite the robust increase in the expression of GLUT4, expression of intracellular glucose-regulated proteins was not altered by either sex of mice or genistein treatment, including AMPK. The effects of genistein on AMPK, a key regulator in glucose metabolism, including glucose uptake, should not be ruled out despite our results. Indeed, future studies are needed to understand the effects of genistein on AMPK activation in conditions associated with insulin resistance, hyperglycemia, and cardiac stress, including reperfusion following ischemia, when glucose metabolism is compromised. Given the pleiotropic nature of genistein, our findings provide important insights in delineating the physiological effects of genistein in the heart.

## Figures and Tables

**Table 1:** The effects of a 4-week genistein diet on echocardiographic parameters of male and female mice.

	Females		Males	
	0 G	600 G	0 G	600 G
End body weight	18.77 ± 1.15	18.24 ± 1.09	25.32 ± 1.86	24.98 ± 1.94
Heart weight	0.114 ± 0.016	0.103 ± 0.005	0.128 ± 0.009	0.135 ± 0.016
IVSd	0.098 ± 0.007	0.097 ± 0.007	0.101 ± 0.002	0.101 ± 0.004
IVSs	0.152 ± 0.009	0.161 ± 0.003	0.163 ± 0.007	0.153 ± 0.020
LVIDd	0.293 ± 0.008	0.313 ± 0.034	0.331 ± 0.034	0.300 ± 0.039
LVIDs	0.156 ± 0.023	0.231 ± 0.033^#^	0.243 ± 0.036^*∗*^	0.214 ± 0.028
LVPWd	0.094 ± 0.005	0.098 ± 0.006	0.101 ± 0.006	0.102 ± 0.007
LVPWs	0.143 ± 0.016	0.156 ± 0.013	0.158 ± 0.014	0.149 ± 0.017
FS (%)	46.65 ± 8.03	26.33 ± 7.66^#^	26.86 ± 5.09^*∗*^	28.19 ± 8.11

0 G: genistein-free diet, 0 mg genistein/kg diet; 600 G: genistein-containing diet, 600 mg genistein/kg diet. All weights are in grams. Values are reported as mean ± SD. *n* = 6–8/group. *∗* denotes significant difference, *P* < 0.05, compared to female 0 G, and # denotes significant difference, *P* < 0.05, genistein-dependent effect.

IVSd: diastolic interventricular wall thickness; IVSs: systolic interventricular wall thickness; LVIDd: diastolic LV internal dimension; LVIDs: systolic LV internal dimension; LVPWd: diastolic LV posterior wall thickness; LVPWs: systolic LV posterior wall thickness; FS: fractional shortening.

## Abbreviations

GLUT4: Glucose transporter 4
CFTR: Cystic fibrosis transmembrane conductance regulatory protein
IVSd: Diastolic interventricular wall thickness
IVSs: Systolic interventricular wall thickness
LVIDd: Diastolic LV internal dimension
LVIDs: Systolic LV internal dimension
LVPWd: Diastolic LV posterior wall thickness
LVPWs: Systolic LV posterior wall thickness
FS: Fractional shortening
iNOS: Inducible nitric oxide synthase
eNOS: Endothelial nitric oxide synthase
MEF2: Myocyte enhancer factor 2
AMPK: AMP-activated protein kinase.
